# Socioeconomic and clinical factors associated with odontogenic infection types among hospitalised patients in Periurban Ghana

**DOI:** 10.1371/journal.pone.0360737

**Published:** 2026-10-12

**Authors:** Kwame Adu Okyere Boadu, Lydia Sarponmaa Asante, Paul Frimpong, Elijah Kwegyir Johnson, Victor Wireko Adu, Richard Okyere Boadu

**Affiliations:** 1 Department of Public Health, School of Public Health and Allied Sciences, Catholic University of Ghana, Fiapre, Ghana; 2 Sunyani Teaching Hospital, Sunyani, Ghana; 3 Department of Health Policy, Management and Economics, School of Public Health, College of Health Sciences, Kwame Nkrumah University of Science and Technology, Kumasi, Ghana; 4 Department of Health Information Management, School of Allied Health Sciences, College of Health and Allied Health Sciences, University of Cape Coast, Cape Coast, Ghana; University of Puthisastra, CAMBODIA

## Abstract

**Background:**

Odontogenic infections may progress to life-threatening complications if not managed promptly, and socioeconomic circumstances have been linked to their presentation, particularly where access to dental care is limited. Because every patient studied was already hospitalised, this analysis addresses the clinical type of infection at presentation, not the risk of developing one.

**Methodology:**

A retrospective cross-sectional review of hospital records was undertaken at the Allied Ward of Sunyani Teaching Hospital (January 2021 to January 2025). Of 811 consecutive admissions, 525 were excluded: 509 for non-odontogenic conditions and 16 for records missing more than 30% of required clinical fields, leaving 286 complete records. Chi-square tests were reported with Cramer’s V and Monte Carlo exact p-values where cells were sparse. Because the unpenalised model showed quasi-complete separation, the primary analysis was a Firth-penalised multinomial logistic regression, with abscess as the reference outcome.

**Results:**

Adjusting for age, sex, employment, residence, recorded oral hygiene, systemic comorbidity and alcohol use, unemployment was associated with higher adjusted odds of Ludwig’s angina (LA) and not abscess (AOR = 2.78, 95% CI 1.27–6.09), and rural residence with necrotizing fasciitis (NF; AOR = 3.62, 95% CI 1.33–9.80). Systemic comorbidity was associated with LA (AOR = 4.14, 95% CI 1.83–9.36), NF (AOR = 8.72, 95% CI 2.95–25.75) and cellulitis (AOR = 2.07, 95% CI 1.03–4.18). Absence of a recorded poor-hygiene entry was associated with cellulitis (AOR = 8.05, 95% CI 2.42–26.83), interpreted as a documentation artefact not a protective effect. Sex and alcohol use showed no association. Number of children, the sole source of separation, was not retained; its penalised intervals included 1.

**Conclusion:**

Unemployment and systemic comorbidity were associated with more severe presentations, and rural residence with NF specifically. Findings are associational and describe variation in infection type among admitted patients rather than infection risk, within a retrospective single-centre design.

## Introduction

Odontogenic infections can be severe and, if not diagnosed and managed promptly, may progress to life-threatening complications. However, the current situation in resource-constrained settings is not reflective of proactive treatment from patients. They normally delay in seeking care [1], which invariably leads to increased severity of the infection and, as such, morbidity and mortality rates increase [2].

Recent studies more often propose the hypothesis that the life course approach is relevant in the research of oral health. Several Structural Equation Modelling studies [3] present evidence of multidirectional relations between life course conditions and adult oral health outcomes, advancing understanding of possible processes through which social determinants operate across the life course [3] or interacting with health impacts across the life course. This has been particularly useful because disentangling direct and indirect effects of early-life SES on oral health outcomes is not straightforward if there are intermediate confounders [4]. Furthermore, longitudinal studies have ascertained the socioeconomic status of a family during the childhood of an individual plays a key role in how the individual perceives oral health beliefs in adolescence and adulthood, and this invariably affects their oral health practices and behaviours at large [4].

Even though the association of early life SES and oral health practices is crystal, this assertion is population and demographic dependent. While some studies conclude that adult SES spearheads current oral health practices and outcomes including odontogenic infections [5, 6]; [7], many others also postulate that the effects of early life SES is only transient and has no real lasting impacts [7]. The influence of oral health on quality of life goes beyond just physical functioning to affect psychological well-being and social interactions. Problems with mastication, swallowing, speech, and the aesthetics of smile due to oral conditions may consequently have effects on self-esteem, social relationships, and, in general, the state of enjoying life [8].

In resource-constrained settings, however, there are inherent problems like limited access to dental care and services as well as economic burdens [9]. As such, there is a heavy burden that results from these infections, leading to high morbidity and mortality [2]. Factors like residence, access to dental care, and complications are key. In these places, preventive therapy is not well prioritized; thus, negative behaviours like poor oral hygiene and substance abuse worsen susceptibility [9].

Delayed care-seeking behaviour leads to swelling, pain, trismus and general malaise [1]. Also, not tackling this head-on leads to heightened cost and complications, which perhaps could have been prevented. Apart from this delay in seeking care, most patients have bad oral health practices [2]. This is further compounded by high smoking rates and underlying dental pathologies [10]. The prognosis is exacerbated with comorbidities and socioeconomic problems. Invariably, there is undue pressure on the healthcare system. Mostly, the vulnerable and medically compromised patients are at the receiving end of this mishap.

While earlier studies superficially treated socioeconomic factors and other clinical factors, this research took a step further to investigate the role of poverty, oral hygiene and systemic comorbidities in determining the clinical type of odontogenic infection (abscess, cellulitis, Ludwig’s angina, or necrotizing fasciitis) diagnosed among admitted patients in Sunyani, Ghana. Accordingly, we hypothesised that socioeconomic factors are associated with the clinical type of odontogenic infection with which patients present at admission, rather than with a single composite treatment outcome.

## Methods

### Study area

This research was carried out in the allied ward, ward designate for dental inpatients in the Sunyani Teaching Hospital (STH), Sunyani. Sunyani East is the exact location of the hospital. Even though the hospital was built in 2003, it was until November 2023 that it was converted to a tertiary facility.

### Study design and type

A retrospective cross-sectional study was done for this study. Data of inpatients who were admitted at the Allied Ward from 2021–2025 on account of odontogenic infections were extracted from the Lightwave Health Information Management System (LHIMS) and ward admission register on 1^st^ April 2025. Authors did not have access to information that could identify individual participants during or after data collection. The diagnosis of an odontogenic infection and its clinical type (abscess, cellulitis, Ludwig’s angina, or necrotizing fasciitis) was the diagnosis recorded by the treating oral and maxillofacial surgery team. The odontogenic origin was established for every patient from the clinically identified offending tooth and the underlying dental condition (dental caries, periodontal disease, retained roots, dental trauma, or failed endodontic treatment), and the affected fascial spaces were documented; infections of non-odontogenic origin fell outside the eligible subpopulation and were not admitted to the analysis. This observational study was designed and is reported in accordance with the STROBE (Strengthening the Reporting of Observational Studies in Epidemiology) guidelines for cross-sectional studies. Because cellulitis, Ludwig’s angina and necrotizing fasciitis form a clinical continuum, assignment to mutually exclusive categories followed an explicit hierarchy applied by the treating oral and maxillofacial surgery team and verified against the operative and discharge notes during abstraction. Each patient was assigned the single most severe category documented during the admission, in the order necrotizing fasciitis > Ludwig’s angina > cellulitis > abscess. Necrotizing fasciitis required documented necrosis of fascia or skin with or without crepitus and a surgical finding of non-viable fascia at debridement. Ludwig’s angina required bilateral submandibular with sublingual and submental space involvement and a documented firm, non-fluctuant swelling with tongue elevation or airway compromise. Cellulitis required diffuse, non-fluctuant fascial-space swelling without a drainable collection and abscess required a localised, fluctuant collection from which pus was drained. Where the admission and final diagnoses differed, the final diagnosis at discharge — that is, the diagnosis after operative findings were available — was used. This is because it reflects the verified extent of the infection. A patient who progressed during admission was therefore classified by the most severe verified presentation, and no patient contributes to more than one outcome category.

### Study population

The study population involved all in-patients admitted to the allied ward of STH from January 2021 to January 2025. From the data, there were 811 admitted patients on account of variants of head and neck injuries and associated diseases.

### Sampling technique and sample size

#### Sampling procedure.

A consecutive census of all eligible cases was used in this study; that is, every admitted patient whose record met the inclusion criteria was included. The records of the 811 admitted persons were analysed to see which ones met the inclusion criteria. Per the study variables, records were extracted. However, neither a peculiar sampling nor exclusion of eligible cases were done. This was to ensure that every patient record meeting the inclusion criteria was well analysed and taken into consideration. In the long run, generalizability and adequate representation was achieved.

### Inclusion criteria

i Patients who are diagnosed with odontogenic infections.
ii Patients admitted at the allied ward of STH from January 2021-January 2025.
iii Patients whose hospital records were sufficiently complete for the study variables (defined a priori as no more than 30% of the required clinical fields missing).

### Exclusion criteria

i Patients who had more than 30% of their clinical records missing.

### Study size

A consecutive census of the eligible subpopulation yielded 286 records from 811 admissions to the Allied Ward between January 2021 and January 2025. Participant flow was as follows. Of the 811 admitted records screened, 525 were not eligible and are accounted for in two separate groups: (i) 509 admissions were for non-odontogenic conditions — head and neck cancers, head injuries, facial and scalp lacerations, diseases of the eye, ear, nose and throat, and admissions for elective surgical procedures — and were therefore outside the eligible subpopulation; and (ii) 16 records were of patients with an odontogenic infection whose chart was missing more than 30% of the required clinical fields and were excluded under the a priori completeness criterion. The remaining 286 records constituted the analytic sample. Because every variable entered into the regression was completely recorded in these 286 charts (Table 4), the regression sample size was also 286 (100% of the analytic sample); no observations were dropped by listwise deletion. With 286 records distributed across four outcome categories, the smallest group (necrotizing fasciitis, n = 44) provided a limited number of events per predictor; estimates involving sparse cells are therefore reported with caution and interpreted alongside their confidence intervals (see Limitations).

### Study variables and measurements

Exposures were the socioeconomic and clinical characteristics recorded in the hospital chart, each operationalised as follows. Age at admission was recorded in years and originally banded into eight categories; for regression it was collapsed into five bands (≤30, 31–40, 41–50 [reference], 51–60 and ≥61 years). Sex was recorded as male or female. Employment status was coded from the occupation field of the admission record as employed if any income-generating occupation was documented (including self-employment, farming, trading and salaried work) and unemployed if the field recorded no occupation, “unemployed”, “housewife” or “student”. The records did not permit grading of occupational class, income or job security. The variable is therefore a coarse binary indicator of documented economic activity. Residence was classified from the recorded home address as urban if the address fell within the Sunyani East or Sunyani West municipal built-up areas, and rural otherwise. The classification followed the Ghana Statistical Service locality designation and reflects administrative locality, not measured travel time to the hospital. Housing tenure was recorded as own property or rented. Number of children was the number of living children recorded at admission (0, 1, 2, 3, 4 or more). Oral hygiene was abstracted from the clinical examination note and is reported here as the presence or absence of a recorded poor-hygiene entry. This distinction is important. The variable separates charts in which the examining clinician explicitly documented poor oral hygiene (n = 251) from charts in which no such entry was made (n = 35). The latter group is not equivalent to verified good oral hygiene, because no standardised plaque or gingival index was applied and the absence of an entry may reflect incomplete documentation rather than a favourable clinical finding. The variable is accordingly labelled “no poor-hygiene entry recorded” rather than “good oral hygiene”. Systemic comorbidity was a binary indicator of any documented chronic condition. Its composition is reported in Table 1 and comprised diabetes mellitus (n = 129, 45.1%), hypertension (n = 68, 23.8%) and HIV/AIDS (n = 27, 9.4%). These categories are not mutually exclusive: 48 patients had more than one documented condition, so the category counts overlap and must not be summed. Substance use was recorded as alcohol only, smoking only, both, or no recorded use The routine record did not permit a reliable distinction between use and clinically defined abuse.

**Table 1:** Distribution of socio-economic factors among selected patients.

Factor	Frequency (N = 286)	Percentage (%)
***Primary Diagnosis***		
Ludwig’s Angina	64	22.4
Necrotizing Fasciitis	44	15.4
Cellulitis	103	36.0
Abscess	75	26.2
***Gender***		
Male	134	46.9
Female	152	53.1
***Employment Status***		
Unemployed	113	39.5
Employed	173	60.5
***History of substance use (as recorded; no entry for 172 patients)***		
Alcohol only	90	31.5
Smoking only	14	4.9
Both	10	3.5
No recorded use	172	60.1
***Systemic comorbidities (e.g., Diabetes, hypertension, HIV) — composition shown below; categories are NOT mutually exclusive***		
No	124	43.4
Yes	162	56.6
Diabetes mellitus	129	45.1
Hypertension	68	23.8
HIV/AIDS	27	9.4
(48 patients had more than one condition; percentages are of N = 286 and sum to more than 56.6%)		
***Poor oral hygiene entry recorded in chart (absence of entry is not verified good hygiene)***		
No entry recorded	35	12.2
Poor hygiene recorded	251	87.8
***Residence Type***		
Rural	160	55.9
Urban	126	44.1
***Number of Children***		
Childless	14	4.9
One child	26	9.1
Two children	85	29.7
Three children	78	27.3
Four or more children	83	29.0
**Housing tenure**		
Own property	101	35.3
Rented	185	64.7
**Routine dental visit reported**		
Yes	19	6.6
No	267	93.4
**Insurance status**		
NHIS	200	69.9
Private insurance	40	14.0
None/ not recorded	46	16.1
**Hospital length of stay (days)**		
1–3	26	9.1
4–7	83	29.0
8–11	77	26.9
12–15	74	25.9
16 or more	26	9.1

*Source: hospital records, Sunyani Teaching Hospital, January 2021 – January 2025 (N = 286).*

### Data collection tool and techniques

Data was extracted using an extraction tool capturing number of days on admission, type of infection and total cost. The extraction tool was implemented on both Google Forms and Microsoft Excel.

### Quality control

Robust measures were enforced at every stage of the work from pre-testing of extraction tool to completed extraction tool editing and data entry. This was to ensure data accuracy and correctness. Completed data were double checked before the next upload starts. At the end of every extraction day, data uploaded by Person A, was crosschecked by Person B and so forth, before the data for the day was secured on clouds.

### Data entry and processing

All data were exported from google forms to Excel for data cleaning. After cleaning the data, they were further saved and transferred to SPSS IBM Version 27 for analysis.

### Data analysis

The socioeconomic and clinical factors assessed were age, sex, employment status, residence, housing tenure, number of children, recorded substance use, recorded oral-hygiene status and systemic comorbidity. Frequencies were tabulated and distributions summarised. The association between each factor and infection type was first examined with a Pearson chi-square test of independence on the full cross-tabulation of factor by infection type (Table 2). Before relying on the chi-square approximation we inspected the expected cell counts of every table and report, for each factor, the minimum expected count and the percentage of cells with an expected count below five. Where that percentage exceeded 20%, or the minimum expected count fell below five, we additionally computed a Monte Carlo exact p-value based on 10,000 replicates (seed 20250915) and report it alongside the asymptotic value. Effect size is reported as Cramer’s V, which is bounded between 0 and 1 for contingency tables of any dimension. The substance-use p-value of 0.060 is the omnibus test for the four-level substance-use variable (alcohol only, smoking only, both, no recorded use) and is not an alcohol-specific test; because 43.8% of cells in that table had an expected count below five, the corresponding Monte Carlo exact p-value is 0.058. It is reported and interpreted as such. For the adjusted analysis, covariates were selected on clinical and epidemiological grounds rather than by bivariate significance screening. The pre-specified set comprised age band and sex (a priori demographic confounders, retained irrespective of significance), employment status and residence (the socioeconomic exposures of primary interest), systemic comorbidity (an established determinant of infection severity), recorded oral-hygiene documentation (the local exposure of interest) and recorded alcohol use (biologically plausible through effects on salivary function and neutrophil activity). Number of children was not retained in the primary model. No mechanism links parity to the anatomical extent of an odontogenic infection independently of household economics, which employment status already captures, and it was the sole source of the separation described below. It is retained in sensitivity analyses. The unpenalised maximum-likelihood multinomial model including number of children exhibited quasi-complete separation. Convergence diagnostics are reported in full in S1 Statistical Analysis and are summarised here. The model reached the iteration limit with the largest absolute coefficient exceeding 13 on the log-odds scale, and the number-of-children x necrotizing fasciitis odds ratios diverged to the order of 10^5–10^6 with standard errors of the same order (the precise values are not identified: they shift with the optimiser stopping point, which is itself a symptom of the separation). The cause was a single empty cell. None of the 14 childless patients presented with necrotizing fasciitis. Because a divergent term can distort the remaining estimates and their standard errors, marking those cells “not estimable” was not a sufficient remedy. The primary analysis is therefore a Firth-penalised multinomial logistic regression, fitted by mean bias reduction (brglm2::brmultinom in R), with abscess as the reference outcome. Firth penalisation yields finite estimates and standard errors under separation and reduces small-sample bias. In the final primary model the largest absolute coefficient was 2.17, all standard errors were finite, and the largest variance inflation factor was below 1.6, indicating neither separation nor material collinearity. Model-level inference used a likelihood-ratio test against the intercept-only model, and each term was tested with a Type II likelihood-ratio test (Table 3 footnote). Nine pre-specified sensitivity analyses (S1 to S9) were run to establish that the substantive conclusions do not depend on the estimator or on the covariate set: the primary penalised model; the identical covariate set fitted by unpenalised maximum likelihood; penalised and unpenalised models with number of children added; a model with the hygiene term removed; a model excluding the 35 patients whose record carried a good-hygiene entry (analytic n = 251); a model in which comorbidity was re-derived from the named conditions rather than the summary field; a model with admission year added; and a parsimonious model. Results appear in Table 6 and S1 Statistical Analysis. Length of stay was recorded in bands and, for the purpose of summarising it, was assigned band midpoints (1–3 days = 2, 4–7 = 5.5, 8–11 = 9.5, 12–15 = 13.5, 16 or more = 16); differences across groups were tested with the Kruskal-Wallis test. This midpointing is an approximation and is noted in the Limitations. Missingness is reported variable by variable with denominators in Table 4. All variables entered into the primary regression were completely recorded, so the analysis was not in fact reduced by listwise deletion and the regression sample size equals the analytic sample size of 286. The priori exclusion of charts missing more than 30% of the required clinical fields took place at the eligibility stage and is described under Study Size. No imputation was undertaken. Descriptive tabulation was performed in IBM SPSS Statistics 27; chi-square tests with Cramer’s V and Monte Carlo p-values, the multinomial models, the penalised fits and all diagnostics were computed in R version 4.x using the nnet, brglm2, MASS and vcd packages.

**Table 2:** Association between socioeconomic and clinical factors and type of odontogenic infection, chi-square tests of independence with Cramer’s V (N = 286).

Factor	χ^2^(df)	P-value (2-tailed)	Cramer’s V
Sex	1.58 (3)	0.665	0.074
Age category (8 bands, as originally analysed)	65.74 (21)	<0.001*	0.277
Employment status	8.25 (3)	0.041*	0.170
Substance use (omnibus, 4 levels)	16.37 (9)	0.060	0.138
Poor-hygiene entry recorded	38.05 (3)	<0.001*	0.365
Residence type	21.22 (3)	<0.001*	0.272
Number of children	21.90 (12)	0.039*	0.160
Systemic comorbidity	46.09 (3)	<0.001*	0.401
Insurance status	23.22 (6)	<0.001*	0.202
Routine dental visit reported	21.66 (3)	<0.001*	0.275
Housing tenure	1.62 (3)	**0.656**	0.075

* significant at the 5% level.

**Table 3:** Firth-penalised multinomial logistic regression of socioeconomic and clinical factors on type of odontogenic infection (N = 286; abscess as the reference outcome).

Predictor (reference)	Ludwig’s Angina	Necrotizing Fasciitis	Cellulitis
	*AOR (95% CI)*	*AOR (95% CI)*	*AOR (95% CI)*
***Age category (ref: 41–50 years)***			
≤30 years	1.57 (0.29–8.66)	1.77 (0.29–10.67)	2.49 (0.63–9.81)
31–40 years	0.23 (0.07–0.75)*	0.14 (0.03–0.68)*	0.64 (0.25–1.61)
51–60 years	0.71 (0.24–2.09)	0.42 (0.13–1.42)	0.50 (0.17–1.42)
≥61 years	1.07 (0.39–2.94)	0.79 (0.27–2.37)	0.88 (0.33–2.32)
***Sex (ref: female)***			
Male	1.78 (0.85–3.71)	1.27 (0.55–2.92)	1.29 (0.67–2.48)
***Employment (ref: Employed)***			
Unemployed	2.78 (1.27–6.09)*	1.34 (0.55–3.26)	1.64 (0.80–3.33)
***Residence (ref: Urban)***			
Rural	0.68 (0.32–1.43)	3.62 (1.33–9.80)*	0.66 (0.34–1.28)
***Oral hygiene documentation (ref: poor hygiene recorded)***			
No poor-hygiene entry recorded	0.99 (0.17–5.67)	1.14 (0.14–9.16)	8.05 (2.42–26.83)***
***Systemic comorbidities (ref: None)***			
Present	4.14 (1.83–9.36)***	8.72 (2.95–25.75)***	2.07 (1.03–4.18)*
***Recorded alcohol use (ref: no recorded use)***			
Recorded	0.96 (0.44–2.09)	1.40 (0.60–3.26)	0.84 (0.41–1.69)

***AOR = adjusted odds ratio; CI = confidence interval; abscess = reference outcome.***

**Table 4:** Completeness of every variable used in the study, with denominators (N = 286).

Variable	Recorded n (%)	Not recorded n (%)	Note
Primary diagnosis (outcome)	286 (100.0)	0 (0.0)	Complete
Age at admission	286 (100.0)	0 (0.0)	Complete
Sex	286 (100.0)	0 (0.0)	Complete
Employment status	286 (100.0)	0 (0.0)	Complete
Residence (urban/rural)	286 (100.0)	0 (0.0)	Complete
Housing tenure	286 (100.0)	0 (0.0)	Complete
Number of children	286 (100.0)	0 (0.0)	Complete
Oral-hygiene entry	286 (100.0)	0 (0.0)	Every chart classified as poor-hygiene recorded (251) or no entry (35)
Systemic comorbidity (yes/no)	286 (100.0)	0 (0.0)	Complete
Named comorbid condition	168 (58.7)	118 (41.3)	Not recorded only where comorbidity = No (118 of 124); 6-patient discrepancy with the summary field, see Limitations
Routine dental visit	286 (100.0)	0 (0.0)	Complete
Hospital length of stay	286 (100.0)	0 (0.0)	Banded; midpoints used
History of substance use	114 (39.9)	172 (60.1)	Analysed as recorded use versus no entry; not verified absence of use
Insurance scheme	240 (83.9)	46 (16.1)	Analysed as a three-level variable including a not-recorded category
Treatment outcome	285 (99.7)	1 (0.3)	Not used as an analytic variable
All variables in the primary regression	286 (100.0)	0 (0.0)	Regression n = 286; no observations dropped by listwise deletion

### Ethical considerations

The Catholic University of Ghana’s Ethical Review Board provided ethical clearance (CUG-ERB-122/25/4-PG) for this study. The ethical clearance together with the introductory letter from the department was sent to the Acting Medical Director’s office of STH. It was also submitted to the Head of Department of the DEENT Sub Budget Management Committee (BMC). Confidentiality was assured and ensured throughout the study. The requirement for individual informed consent was explicitly waived by the Catholic University of Ghana Ethical Review Board in its approval of this protocol, on the grounds that the study used only pre-existing, routinely collected secondary clinical records and that no patient contact took place. The authors did not have access to information that could identify individual participants during or after data collection. Records were de-identified at abstraction and analysed under a study number only (Table 5).

**Table 5:** Insurance status and infection type (N = 286).

Insurance status	n (%)	Median LOS (days)	IQR (days)
**NHIS**	**200 (69.9)**	**9.5**	**5.5–13.5**
**Private insurance**	**40 (14.0)**	**5.5**	**5.5–9.5**
**None/ not recorded**	**46 (16.1)**	**13.5**	**5.5–16.0**

IQR = interquartile range; NHIS = National Health Insurance Scheme. Insurance status was associated with infection type (chi-square = 23.22, df = 6, p < 0.001; Cramer’s V = 0.202).

## Results

The study examined how socio-economic (SE) factors such as gender, age, employment status, history of substance use, evidence of poor hygiene, presence of systemic comorbidities, influence one’s chances of getting affected by an odontogenic infection. As shown in Table 1, the distribution of gender was dominated by females – 152(53.1%) indicating that many females were affected by odontogenic infections than the males sampled. Also, people who were employed had more number of infections per the distribution – 173(60.5%), alcohol abusers were mostly affected among those who had a history of substance abuse – 90(31.5%). Similarly, people with systemic comorbidities also had more recorded infections than those without it. Also, many of the sampled had an evidence of poor hygiene −251(87.8%).

The association between each socioeconomic factor and infection type was examined with chi-square tests of independence (Table 2), reported with Cramer’s V as the effect size, the minimum expected cell count, the percentage of cells with an expected count below five, and a Monte Carlo exact p-value where the table was sparse. Age category (chi-square = 65.74, df = 21, p < 0.001; Monte Carlo p < 0.001; Cramer’s V = 0.277), employment status (chi-square = 8.25, df = 3, p = 0.041; V = 0.170), recorded poor oral hygiene (chi-square = 38.05, df = 3, p < 0.001; V = 0.365), residence type (chi-square = 21.22, df = 3, p < 0.001; V = 0.272), number of children (chi-square = 21.90, df = 12, p = 0.039; Monte Carlo p = 0.037; V = 0.160), systemic comorbidity (chi-square = 46.09, df = 3, p < 0.001; V = 0.401), insurance status (chi-square = 23.22, df = 6, p < 0.001; V = 0.202) and reported routine dental visit (chi-square = 21.66, df = 3, p < 0.001; Monte Carlo p < 0.001; V = 0.275) were each associated with infection type. Sex (chi-square = 1.58, df = 3, p = 0.665; V = 0.074) and housing tenure (chi-square = 1.62, df = 3, p = 0.656; V = 0.075) were not. The overall substance-use variable was not associated with infection type (chi-square = 16.37, df = 9, p = 0.060; Monte Carlo p = 0.058; V = 0.138); this omnibus p-value refers to the four-level substance-use variable and is not an alcohol-specific test. Cramer’s V values ranged from 0.074 to 0.401, indicating weak to moderate associations. The age and routine-dental-visit tables were sparse (37.5% of cells with an expected count below five) and the substance-use table more so (43.8%); for these the Monte Carlo exact p-values, which do not rely on the chi-square approximation, are the values we interpret. Adjusted associations were then estimated in a Firth-penalised multinomial logistic regression with a clinically specified covariate set (Table 3); sex and recorded alcohol use were retained as a priori covariates despite their null bivariate associations.

In the Firth-penalised multinomial model (Table 3; abscess as the reference outcome), unemployment was associated with higher adjusted odds of presenting with Ludwig’s angina rather than abscess (AOR = 2.78, 95% CI 1.27–6.09, p = 0.011), as was systemic comorbidity (AOR = 4.14, 95% CI 1.83–9.36, p < 0.001). Relative to the 41–50-year reference band, patients aged 31–40 years had lower adjusted odds of Ludwig’s angina (AOR = 0.23, 95% CI 0.07–0.75, p = 0.015). No other age band reached conventional significance. Male sex was not associated with Ludwig’s angina (AOR = 1.78, 95% CI 0.85–3.71, p = 0.127), nor was rural residence (AOR = 0.68, 95% CI 0.32–1.43) or recorded alcohol use (AOR = 0.96, 95% CI 0.44–2.09) (Fig 1).

**Fig 1:**
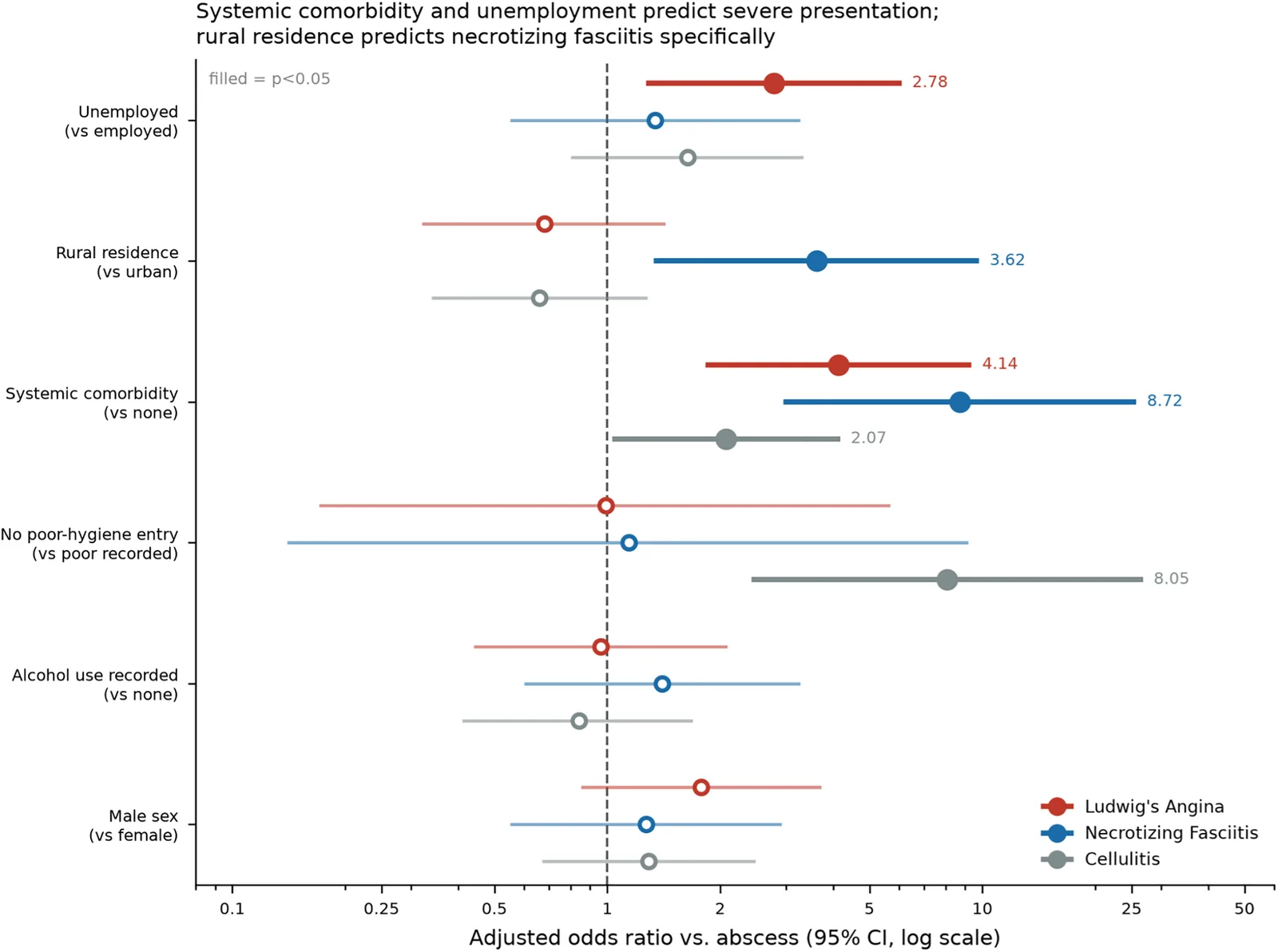
Adjusted odds ratios from the Firth-penalised multinomial model, by infection type relative to abscess. Markers show adjusted odds ratios and horizontal lines 95% confidence intervals on a logarithmic scale; filled markers indicate p < 0.05. Systemic comorbidity and unemployment are associated with the more severe presentations, and rural residence with necrotizing fasciitis specifically (Figure 1).

For necrotizing fasciitis, rural residence (AOR = 3.62, 95% CI 1.33–9.80, p = 0.012) and systemic comorbidity (AOR = 8.72, 95% CI 2.95–25.75, p < 0.001) were associated with substantially higher adjusted odds relative to abscess, and patients aged 31–40 years had lower odds than the 41–50-year band (AOR = 0.14, 95% CI 0.03–0.68, p = 0.015). Employment status (AOR = 1.34, 95% CI 0.55–3.26), male sex (AOR = 1.27, 95% CI 0.55–2.92) and recorded alcohol use (AOR = 1.40, 95% CI 0.60–3.26) were not associated with necrotizing fasciitis. In the unpenalised model including number of children, the corresponding odds ratios for necrotizing fasciitis diverged to the order of 10^5–10^6 because no case of necrotizing fasciitis occurred among the 14 childless patients (Fig 2, left panel). Under Firth penalisation the same terms were finite and all confidence intervals included 1 (one child AOR = 4.75, 95% CI 0.14–164.55; two children AOR = 1.89, 95% CI 0.06–57.60; three children AOR = 2.77, 95% CI 0.09–81.17; four or more AOR = 5.61, 95% CI 0.19–168.05; Fig 2, right panel, and sensitivity analysis S3). Number of children is not part of the primary covariate set for the reasons given in the Data Analysis section.

**Fig 2:**
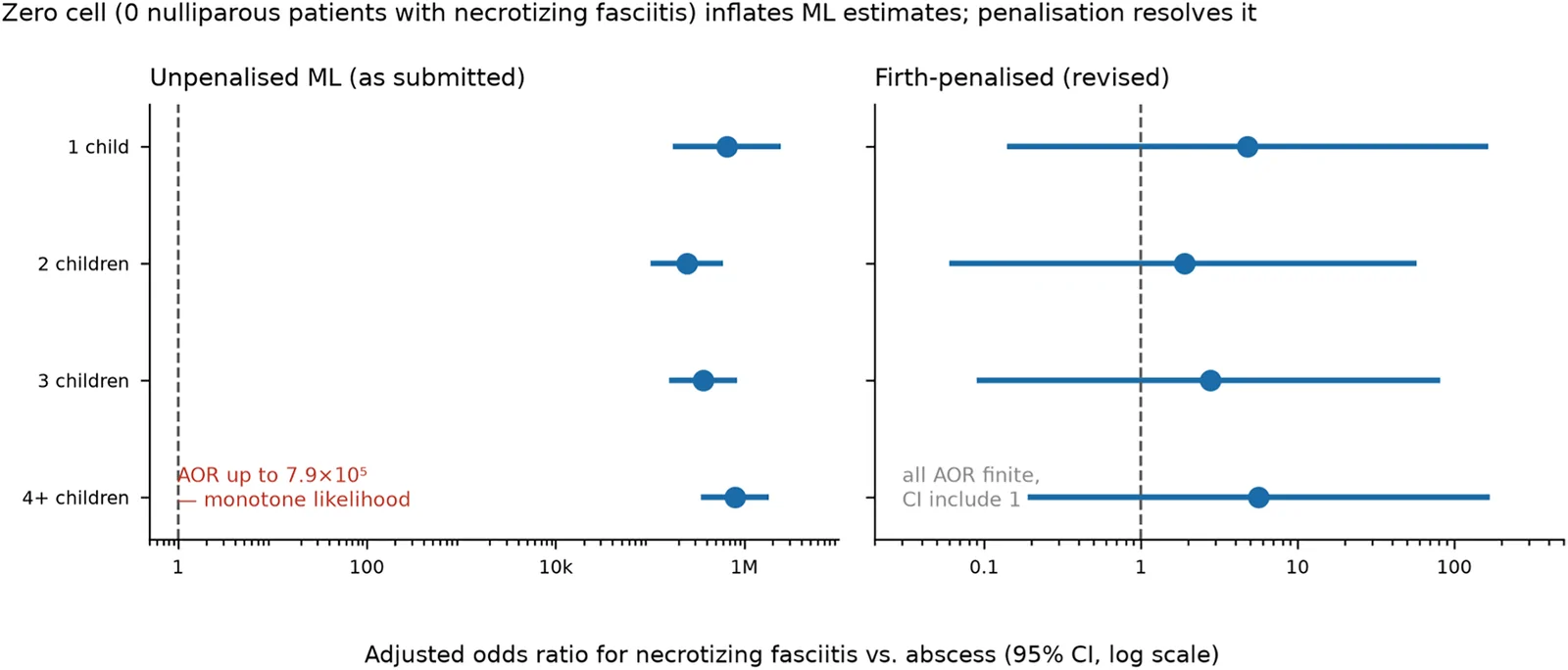
Effect of quasi-complete separation on the number-of-children estimates, and its resolution by penalisation. Left panel: unpenalised maximum-likelihood estimates, in which the number-of-children odds ratios for necrotizing fasciitis diverge to the order of 10^5–10^6 because no case of necrotizing fasciitis occurred among the 14 childless patients. Right panel: Firth-penalised estimates for the same terms, which are finite with all confidence intervals including 1.

For cellulitis, no age band was independently associated with the outcome after adjustment and collapsing of the age categories, and systemic comorbidity was associated with modestly higher adjusted odds (AOR = 2.07, 95% CI 1.03–4.18, p = 0.042). The strongest association was with the oral-hygiene documentation variable. Patients with no recorded poor-hygiene entry had higher adjusted odds of cellulitis than patients in whom poor hygiene was explicitly documented (AOR = 8.05, 95% CI 2.42–26.83, p < 0.001). This estimate denotes higher odds of cellulitis relative to abscess, not a protective association with respect to infection; it is examined in the Discussion.

The direction of this estimate warrants emphasis. An adjusted odds ratio above 1 for the no-poor-hygiene-entry group indicates that those patients were more likely, not less likely, to present with cellulitis rather than abscess. The estimate rests on 35 patients (12.2%), and documentation bias and sparse data are plausible explanations for it; neither is demonstrated by these data.

Rural residence was not associated with cellulitis after adjustment (AOR = 0.66, 95% CI 0.34–1.28), nor was unemployment (AOR = 1.64, 95% CI 0.80–3.33), male sex (AOR = 1.29, 95% CI 0.67–2.48) or recorded alcohol use (AOR = 0.84, 95% CI 0.41–1.69).

The penalised model as a whole was informative relative to the intercept-only model (likelihood-ratio chi-square = 132.32, df = 30, p < 0.001; McFadden pseudo-R-squared = 0.172, Nagelkerke R-squared = 0.398). In Type II likelihood-ratio tests of individual terms, recorded oral hygiene (chi-square = 29.74, df = 3, p < 0.001), systemic comorbidity (chi-square = 25.75, df = 3, p < 0.001), residence (chi-square = 17.73, df = 3, p < 0.001), age band (chi-square = 22.74, df = 12, p = 0.030) and employment (chi-square = 7.97, df = 3, p = 0.047) contributed to fit, whereas sex (chi-square = 2.62, df = 3, p = 0.454) and recorded alcohol use (chi-square = 1.71, df = 3, p = 0.635) did not. Both were nevertheless retained as a priori covariates. The largest absolute coefficient in the model was 2.17 and the largest variance inflation factor was below 1.6, indicating that neither separation nor material collinearity affected the reported estimates. Across the nine sensitivity specifications (Table 6), the associations of unemployment with Ludwig’s angina, of rural residence with necrotizing fasciitis, and of systemic comorbidity with both severe presentations were consistent in direction and magnitude, and the hygiene-documentation association with cellulitis persisted in every specification in which the term was included. Crude odds ratios, full coefficients, convergence diagnostics, goodness-of-fit statistics and multicollinearity diagnostics are provided in S1 Statistical Analysis.

**Table 6:** Sensitivity of the key associations to estimator and covariate set (nine pre-specified specifications; adjusted odds ratios with 95% confidence intervals, abscess as the reference outcome).

Specification	Unemployed → LA	Rural → NF	Comorbidity → LA	Comorbidity → NF	No hygiene entry → Cellulitis
S1 Primary (Firth, clinical covariate set)	2.78 (1.27–6.09)	3.62 (1.33–9.80)	4.14 (1.83–9.36)	8.72 (2.95–25.75)	8.05 (2.42–26.83)
S2 Same covariate set, unpenalised ML	2.94 (1.33–6.51)	4.01 (1.41–11.43)	4.48 (1.96–10.24)	10.48 (3.31–33.21)	9.95 (2.77–35.67)
S3 Firth + number of children	2.33 (1.05–5.19)	4.14 (1.51–11.33)	3.76 (1.57–9.01)	8.49 (2.83–25.50)	7.58 (2.29–25.10)
S4 Unpenalised ML + number of children (separated)	2.49 (1.10–5.61)	4.81 (1.64–14.07)	4.16 (1.71–10.12)	10.85 (3.31–35.52)	9.64 (2.68–34.76)
S5 Firth, hygiene term removed	2.57 (1.18–5.63)	3.37 (1.24–9.13)	4.28 (1.88–9.74)	9.15 (3.08–27.17)	term not in model
S6 Firth, excluding the 35 charts with no hygiene entry (n = 251)	2.47 (1.12–5.48)	4.42 (1.52–12.82)	4.04 (1.77–9.20)	10.89 (3.38–35.10)	term not estimable by design
S7 Firth, comorbidity re-derived from named conditions	2.68 (1.21–5.93)	3.46 (1.28–9.39)	5.82 (2.48–13.64)	8.62 (2.90–25.63)	7.53 (2.27–25.00)
S8 Firth + admission year	2.67 (1.22–5.87)	3.84 (1.41–10.45)	4.03 (1.77–9.20)	9.06 (3.08–26.63)	7.91 (2.37–26.39)
S9 Firth, parsimonious (age, employment, residence, hygiene, comorbidity)	2.80 (1.29–6.07)	3.60 (1.33–9.74)	4.03 (1.81–9.02)	9.11 (3.08–26.94)	8.46 (2.52–28.36)

LA = Ludwig’s angina; NF = necrotizing fasciitis; ML = maximum likelihood. All specifications use abscess as the reference outcome and n = 286 except S6 (n = 251). Each of the five reported associations retains its direction and, apart from the expected inflation under unpenalised estimation in S2 and S4, a similar magnitude across all specifications, so the substantive conclusions do not depend on the choice of estimator or covariate set. In S4 the number-of-children terms for necrotizing fasciitis remain non-identifiable (odds ratios of the order of 10^5–10^6) even though the terms shown here are stable; this is the separation that motivated the penalised primary model. Full output for all nine specifications, including every coefficient and all diagnostics, is provided in S1 Statistical Analysis.

Percentages are of the 286 eligible records. The 16 charts excluded at the eligibility stage for missing more than 30% of required fields are not represented in this table, as they are outside the analytic sample. Participant flow is described in Study Size. No imputation was performed. Because all variables entered into the primary model were completely recorded, the analysis is not a reduced complete-case analysis.

## Discussions

### The role of employment and family dynamics

Employment status was associated with the severity of presentation in this cohort: unemployed patients had higher adjusted odds of Ludwig’s angina than of a localised abscess (AOR = 2.78, 95% CI 1.27–6.09). Employment plausibly operates through income and the capacity to meet the direct and indirect costs of timely dental care, a pathway described in income-and-oral-health reviews [11]. It should be noted that dental only insurance is uncommon in low- and middle-income settings such as Sunyani. In a comparable series of odontogenic infections, abscesses predominated among housewives (31.8%) and workers (27.3%) [12]; in the present study 60.5% (173) of patients were classified as employed. The records did not capture occupation type, income, job security or occupational class, so the mechanism linking employment to presentation cannot be examined here and the binary employment indicator should be read as a coarse marker of documented economic activity only.

The distribution of number of children in this cohort (two children 29.7%, n = 85; four or more 29.0%, n = 83; three children 27.3%, n = 78; one child 9.1%, n = 26; childless 4.9%, n = 14) broadly resembles that reported by Ekici [12]. Number of children was not independently associated with infection type in the penalised sensitivity model in which it was included (all confidence intervals included 1), and it was the sole source of the separation that affected the previously reported model. Household size and parity were not measured in a way that would support inference about budgetary allocation or time available for dental care, and we therefore make no claim about such pathways.

Marital status, household decision-making and care-seeking delay were not recorded in the charts used for this study, and the preceding paragraphs should not be read as evidence about them. Whether childless patients present earlier than parents with odontogenic infections remains an open question that this retrospective design, with only 14 childless patients, cannot answer; a prospective study measuring time from symptom onset to presentation alongside household composition would be required.

### Access to dental care

In managing odontogenic infections, proximity to dental care is a focal point that cannot be overlooked. This linchpin is extensively arbitrated by socioeconomic markers. There are systemic disparities to this effect. In 2008, the total spending amounts for dental care depreciated to 5.3%. In the same vein, that of the USA increased faintly to 7.3%. The glaring disparities between those countries cannot be overlooked though [13]. In Ekici’s study, most of his respondents (72.7%) resided in the urban centres. This study saw opposite findings with 44.1% (126 respondents) of respondents in urban area. Their large proportion of urban residents indicated proximity to the dental clinic [12], which was contrary to this study where more rural residents 55.9% (160 respondents) were realized. In their system, less than 15% of respondents went directly to the oral and maxillofacial surgery (OMFS) clinic. In STH, the OMFS clinic is adjacent to the oral diagnostic clinic where ‘general’ consultation is done. As such, most patients walk into the clinic. Others are brought to the emergency department and reviewed by the on-call OMFS doctor. In Ekici [12], 13.5% of respondents waited for close to two weeks before seeking treatment after symptoms. The reasons were attributed to transport barriers, poverty or awareness issues [9]. While the exact number of days patients waited before reporting is not known in this study due to the nature of the data, the sentiments in Ekici [12] resonates with that of STH OI patients, especially, the majority in rural areas.

It has been established that low income influences the treatment seeking behaviour of patients [11]. Schwendicke et al. [7] on the other hand argue that regardless of access to dental care, disparities still exist. They strongly believe that people with low socioeconomic status tend to adopt a reactive approach to health rather than proactive and preventive care [7]. The urban-rural polarization from Ekici’s point of view is in line with this. For patients in the rural communities, they travel for very long hours to see a dentist. The delays in access can also be attributed to the systemic diseases patients present with. Approximately 40.9% of Ekici’s respondents either had diabetes or hypertension. This was contrary to this study where 56.6% (162) of the respondents had one or more systemic comorbidities (e.g., diabetes, hypertension, HIV). As stated earlier, Ghana is characterised by a strong sense of communal belonging, and patients often share health information within their communities. Most of the comorbid patients say they fail to present to the clinic early because they feel they will be hospitalized for long. As irrational as it appears to be, it is a major reason why patients with OIs present later to the hospital when admission is inevitable.

Insurance coverage in this cohort was as follows: 200 patients (69.9%) were registered with the National Health Insurance Scheme, 40 (14.0%) held private insurance, and for 46 (16.1%) no insurance field was completed (Table 4). Insurance status was associated with infection type in the bivariate analysis (chi-square = 23.22, df = 6, p < 0.001; Cramer’s V = 0.202; Table 2). Dental procedures are covered only to a limited extent under the NHIS benefit package, and the scope of dental coverage in Ghana remains an area warranting formal health-financing research. Whether teledentistry or mobile dental outreach could narrow the access gap for the surrounding rural districts is a question the present data cannot address, but it is a plausible direction for service development and evaluation at the teaching hospital.

### Behavioural factors

Alcohol is a known modifiable risk factor in many pathologies with odontogenic infections, not an exception. It should be noted that abuse of alcohol renders the salivary gland to dysfunction, desiccates the oral mucosa and completely changes the normal oral pH. The aftermath of this is to make the oral environment susceptible to colonization by microbes [14]. In Manicone’s study, it was realized that individuals who abused alcohol comparatively had higher prevalence rates of caries and periodontal diseases. Also, these people demonstrated very poor oral hygiene when compared to their counterparts [14]. In this study, 31.5% (90) of respondents either took alcohol occasionally or abused them. Alcohol has been found to prevent patients from seeking dental care earlier [14].

Delay in seeking dental care means that carious lesions are not treated early, with progressive pulpal involvement and eventual odontogenic infection [14]. Costa et al. [15] reported that patients with longer ward stays were more often alcohol users. In the present study the overall substance-use variable was not associated with infection type (chi-square = 16.37, df = 9, p = 0.060; Monte Carlo p = 0.058; Cramer’s V = 0.138). We emphasise that this p-value is the omnibus test across the four substance-use categories and is not an alcohol-specific result. In the adjusted model, recorded alcohol use was not associated with any infection type (Ludwig’s angina AOR = 0.96, 95% CI 0.44–2.09; necrotizing fasciitis AOR = 1.40, 95% CI 0.60–3.26; cellulitis AOR = 0.84, 95% CI 0.41–1.69). Because 172 patients (60.1%) had no substance-use entry at all, this variable is better understood as recorded use versus no recorded entry than as a measure of true exposure, and the null finding may reflect that measurement limitation rather than an absence of effect.

The role of smoking (tobacco) to the oral cavity is inimical. Its pathophysiology is not complicated. It constricts the blood vessels of the gingivae, disrupts neutrophil function and increases the degree of adhesion of oral biofilm [16]. In the grand scheme of affairs, all the mentioned effects lead to the destruction of the periodontal tissue and ligaments. This invariably increases the risk of odontogenic infections (OIs). A 2012 study saw that the combination of alcohol and smoking created a synergy for the severity of odontogenic infections [17]. Only 3.5% (10) of respondents smoked and took alcohol. Heavy smokers have also been reported to have abysmal oral hygiene practices [16]. This study saw 4.9% (14) respondents as smokers. In the Ghanaian context, smoking is more heavily stigmatised than alcohol use, so the higher number of alcohol users than smokers in this study was not surprising. In fact, some patients are normally shy to admit to any of these behaviours. There is a link between tobacco smoking and subclinical infection [16]. It masks the progression of diseases to make them appear not in critical stages. Odontogenic infections will not come to being if oral hygiene practices are not optimum.

Caries, gingivitis and periodontal disease stem from poor plaque control, and these conditions progress to odontogenic infection when the primary source is not addressed [18]. In this study a poor-hygiene entry was documented in 87.8% (251) of charts, and the presence of such an entry was associated with infection type (chi-square = 38.05, df = 3, p < 0.001; Cramer’s V = 0.365). The role of age in odontogenic infections is double-edged: younger individuals acquire poor oral hygiene largely through lifestyle factors, whereas older individuals face reduced salivary flow and comorbidities that adversely affect oral hygiene. An earlier prospective series found that spread of odontogenic infection was more extensive in older patients [19]. Age was associated with infection type in the bivariate analysis (chi-square = 65.74, df = 21, p < 0.001; Monte Carlo p < 0.001; Cramer’s V = 0.277). In the adjusted model, after collapsing age into five bands, the 31–40-year band had lower odds of both Ludwig’s angina and necrotizing fasciitis than the 41–50-year reference band, while no age band was independently associated with cellulitis.

The association between the absence of a recorded poor-hygiene entry and cellulitis requires careful statement. The adjusted odds ratio of 8.05 (95% CI 2.42–26.83) means that patients whose charts contained no poor-hygiene entry had approximately eight times the odds of presenting with cellulitis rather than with a localised abscess, compared with patients in whom poor hygiene was explicitly documented. It does not indicate that recorded good hygiene protected against infection, and it says nothing about the risk of acquiring an odontogenic infection, since every patient in the study already had one. Two explanations are plausible but neither is demonstrated by these data. The first is differential documentation: patients presenting with diffuse cellulitis are assessed urgently, often by a different clinician and under time pressure, and the oral-hygiene field may be left blank more often in that context than during the more deliberate examination of a patient with a drainable abscess. On this account the variable partly measures who was examined in what circumstances rather than the patient’s hygiene. The second is sparse-data bias: only 35 patients (12.2%) lacked a poor-hygiene entry, and estimates based on cells of that size are imprecise, as the wide confidence interval shows. A third possibility, which we cannot exclude, is misclassification arising from abstraction of hygiene from free-text notes in the absence of a standardised plaque or gingival index. We examined the robustness of the estimate in two ways. Removing the hygiene term left the other associations unchanged (sensitivity analysis S5), and excluding the 35 patients whose record carried a good-hygiene entry (n = 251; sensitivity analysis S6) also left the unemployment, residence and comorbidity associations essentially unchanged. We note explicitly that neither procedure can establish why the hygiene association arose: removing the patients who generate an estimate demonstrates only that the remaining estimates do not depend on them. Distinguishing documentation bias from a genuine clinical relationship would require prospective recording of a standardised hygiene index at presentation, which this retrospective design cannot provide. We therefore report the association, state its direction correctly, and refrain from attributing it to a demonstrated cause.

## Limitations

Some limitations should be considered. First, the design is retrospective and cross-sectional; it identifies associations and cannot establish causation. Second, and fundamental to interpretation, every patient in this study was already hospitalised with an odontogenic infection. The analysis therefore describes which factors are associated with the clinical type of infection among admitted patients; it provides no information about the risk of developing an odontogenic infection, and none of the estimates should be read as risk factors for infection. Third, all patients were drawn from a single tertiary teaching hospital that also serves as a referral centre for the surrounding rural districts, raising the possibility of selection (Berkson-type) bias and over-representation of severe cases; this may partly explain the high proportion of rural residents and limits generalisability to community and primary-care settings. Fourth, exposures including oral hygiene, substance use and systemic comorbidity were abstracted from routine records rather than measured with standardised instruments, and were dichotomised, reducing granularity and permitting misclassification; the oral-hygiene variable in particular distinguishes only the presence or absence of a clinician-recorded poor-hygiene entry and cannot identify verified good hygiene. Fifth, the systemic comorbidity indicator is a composite of diabetes mellitus, hypertension and HIV/AIDS whose categories are not mutually exclusive (48 patients had more than one condition); the composite cannot separate the contributions of the individual conditions, and diabetes in particular is likely to dominate it. Also, sparse data remain a limitation: the smallest outcome group, necrotizing fasciitis, comprised 44 patients, and several covariate cells were small, producing wide confidence intervals. In an unpenalised maximum-likelihood model this sparseness caused quasi-complete separation through a single empty cell (no necrotizing fasciitis among the 14 childless patients), which produced divergent odds ratios; the present analysis addresses this with Firth penalisation and by not retaining number of children in the primary model, and the revised estimates are finite with a maximum absolute coefficient of 2.17. Finally, the study period (2021–2025) spans the COVID-19 pandemic; admission year was added in sensitivity analysis S8 without material change to the estimates, but pandemic-related disruption of care-seeking cannot be fully excluded as an influence on presentation and severity.

## Conclusion

Among patients hospitalised with odontogenic infections at this centre, socioeconomic and clinical factors were associated with the clinical type of infection at presentation. Unemployment was associated with higher adjusted odds of presenting with Ludwig’s angina than with abscess (AOR = 2.78, 95% CI 1.27–6.09), and rural residence with higher adjusted odds of necrotizing fasciitis than urban residence (AOR = 3.62, 95% CI 1.33–9.80). Systemic comorbidity was associated with higher adjusted odds of Ludwig’s angina (AOR = 4.14, 95% CI 1.83–9.36), necrotizing fasciitis (AOR = 8.72, 95% CI 2.95–25.75) and cellulitis (AOR = 2.07, 95% CI 1.03–4.18) relative to abscess. Number of children was not independently associated with infection type and is not part of the primary model; in an unpenalised model its necrotizing-fasciitis estimates were not identifiable because no case of necrotizing fasciitis occurred among childless patients, and under penalisation the corresponding estimates were finite with confidence intervals including 1.

Age was associated with infection type in the bivariate analysis, and in the adjusted model patients aged 31–40 years had lower odds of Ludwig’s angina and necrotizing fasciitis than the 41–50-year reference band, while no age band was independently associated with cellulitis. The absence of a recorded poor-hygiene entry was associated with higher adjusted odds of cellulitis relative to abscess (AOR = 8.05, 95% CI 2.42–26.83). This is an association with higher odds of the more diffuse presentation, not a protective effect; differential documentation and sparse data (35 patients) are plausible explanations that these data cannot confirm. Sex and recorded substance use were not associated with infection type. Systemic comorbidity (56.6%) and a recorded poor-hygiene entry (87.8%) were both common in this cohort. All findings are associational, pertain to infection type among admitted patients rather than to infection risk, and should be interpreted in light of the retrospective, single-centre design. Together they indicate that the socioeconomic and clinical circumstances of patients hospitalised with odontogenic infections are associated with how severely those infections present.

### Recommendations

Future studies can look at how tele dentistry can be harnessed to improve dental health seeking behaviour among both urban and rural dwellers.
